# From drug mechanism–based to driver-guided therapy: targeting disease drivers in psoriatic arthritis

**DOI:** 10.1016/j.ero.2026.100216

**Published:** 2026-07-12

**Authors:** Thorben Witte, Arnd Kleyer, Francesco Ciccia, David Simon

**Affiliations:** 1 Department of Rheumatology and Clinical Immunology, Charité—Universitätsmedizin Berlin, Berlin, Germany; 2 Department of Allergology and Immunology, Fraunhofer Institute for Translational Medicine and Pharmacology, Berlin, Germany; 3 Deutsches Rheuma-Forschungszentrum Berlin, Berlin, Germany; 4 Department of Precision Medicine, Rheumatology Section, University della Campania L Vanvitelli, Naples, Italy

Psoriatic arthritis (PsA) is characterised by profound clinical heterogeneity, involving peripheral arthritis, enthesitis, dactylitis, axial disease, and skin manifestations [1,2]. Accordingly, PsA is increasingly recognised not as a single disease entity but as a spectrum of related inflammatory phenotypes involving multiple tissues and immune pathways. Current treatment decisions already account for major clinical domains and comorbid manifestations, such as prominent skin disease, inflammatory bowel disease, or uveitis, as reflected in contemporary management recommendations. However, this level of stratification remains relatively broad. Despite this complexity, therapeutic decision making in routine practice still largely follows a sequential trial-and-error approach based on drug availability and previous treatment exposure, whereas deeper biological, molecular, and tissue-level characteristics of the individual patient are rarely used to guide treatment selection.

Over the past decade, the therapeutic landscape of PsA has expanded substantially with the introduction of biologic and targeted synthetic disease-modifying antirheumatic drugs, including inhibitors of tumour necrosis factor, interleukin (IL)-17, IL-23, and Janus kinase signalling pathways [3]. While these agents have significantly improved outcomes for many patients, treatment responses remain heterogeneous, and a considerable proportion of patients fail to achieve sustained low disease activity or minimal disease activity [4,5]. Real-world observational studies indicate that a substantial subset of patients cycles through multiple therapeutic mechanisms without achieving durable disease control, highlighting the limitations of the current empirical treatment paradigm [[6], [7], [8]].

This raises a central question for contemporary PsA management: rather than asking which drug should be used next, should we instead ask which disease driver is dominant in an individual patient? A similar conceptual transition has already transformed oncology, where treatment strategies increasingly rely on tumour biology and molecular drivers rather than empirical treatment sequencing [9]. We propose that a comparable shift, from mechanism-based treatment to driver-guided therapy, may represent the next step towards precision medicine in PsA. This viewpoint synthesises emerging evidence from clinical phenotyping, imaging studies, and translational research to outline a conceptual framework for driver-informed treatment stratification in PsA.

## CLINICAL PHENOTYPES OF PsA

Multiple recent studies have applied clustering approaches to characterise clinical phenotypes of PsA. These analyses consistently demonstrate that patients with PsA can be grouped into clinically meaningful clusters defined by the dominance of specific disease domains [10,11].

Machine learning–based clustering analyses of randomised clinical trial populations have identified phenotypes characterised by distinct patterns of joint involvement and extra-articular disease manifestations. In an analysis of patients enrolled in phase III trials of IL-23 inhibition, 5 major clusters were identified, including large joint–dominant disease, enthesitis-predominant disease, hand-dominant arthritis, dactylitis-associated disease, and foot-dominant arthritis [11]. Importantly, although therapeutic responses were observed across clusters, the probability of achieving composite treatment targets such as minimal disease activity varied between phenotypes.

Complementary insights emerge from real-world cohort studies. In a multicentre observational analysis from the Psoriatic Arthritis Research Consortium, clustering methods applied to routine clinical data identified 3 stable phenotypic groups, including an oligoarticular phenotype, a multidomain inflammatory phenotype, and a phenotype characterised by a high burden of patient-reported symptoms. Importantly, these clusters were associated with distinct longitudinal trajectories of disease activity and treatment response, highlighting the potential clinical relevance of phenotype-based stratification in routine care [10].

These findings suggest that clinically observable disease patterns capture important aspects of underlying disease heterogeneity. However, clinical phenotypes alone may not fully capture the dominant drivers of disease activity, emphasizing the need to integrate additional layers of information.

## IMAGING-DEFINED INFLAMMATION AS A KEY DISCRIMINATOR

One important limitation of purely clinical phenotyping approaches is that clinical assessments alone may not accurately capture the inflammatory activity underlying symptoms. Imaging techniques such as musculoskeletal ultrasound and magnetic resonance imaging (MRI) offer the possibility to directly visualise inflammatory lesions and, therefore, refine phenotypic classification [12,13].

This distinction becomes particularly relevant in patients with difficult-to-manage or treatment-refractory PsA [14,15]. In an ultrasound-based study of patients fulfilling criteria for difficult-to-treat PsA, 2 distinct phenotypes were identified: one characterised by persistent imaging-detected synovitis or enthesitis and another in which patients exhibited ongoing symptoms despite the absence of objective inflammatory findings [16]. These observations suggest that clinically defined disease activity may reflect a mixture of inflammatory and noninflammatory drivers, which require fundamentally different therapeutic strategies. The ability to differentiate between inflammatory and noninflammatory drivers of disease activity has important therapeutic implications. In patients with persistent inflammatory lesions, escalation or switching of immunomodulatory therapy may be appropriate. Conversely, in patients without detectable inflammation, alternative drivers such as structural damage, central pain sensitisation, or comorbid conditions may need to be addressed.

Recent advances in imaging further expand this concept. Whole-body MRI studies have demonstrated that treatment-induced improvements in clinical disease activity are not always paralleled by identical patterns of change in imaging-detected inflammation. These findings suggest that imaging may reveal mechanistic differences between therapeutic agents that are not fully captured by clinical composite scores [17].

Beyond ultrasound and MRI, rapid advances in molecular imaging may further refine this approach. Positron emission tomography using tailored tracers has the potential to visualise specific cellular or stromal processes within inflamed tissues, such as activated fibroblasts, macrophage-rich inflammation, vascular activation, or bone-remodelling pathways. These techniques may therefore help move imaging from the detection of inflammation towards the identification of disease drivers [18,19]. Although currently mainly a research tool, molecular imaging could ultimately support patient stratification, response prediction, and mechanism-specific treatment selection in PsA.

Together, these observations indicate that imaging biomarkers have the potential to refine clinical phenotypes and may represent an important step towards more precise treatment stratification in PsA. Thus, imaging may provide an intermediate layer linking clinical phenotypes with underlying inflammatory mechanisms.

## BEYOND INFLAMMATION: MODIFIERS OF TREATMENT RESPONSE

Clinical phenotypes alone are unlikely to fully explain the variability in therapeutic responses observed in PsA. Several additional factors appear to modify disease expression and treatment outcomes.

Real-world studies investigating patients with multidrug refractory PsA have identified several clinical characteristics associated with repeated treatment failure. These include axial disease involvement, obesity, depression, and female sex [6,20]. Importantly, these factors likely influence treatment response through distinct mechanisms, including altered immune signalling, pharmacokinetic variability, and amplification of symptom burden independent of active inflammation.

Obesity represents a particularly relevant modifier. Increasing evidence suggests that excess adiposity contributes to systemic inflammation, mechanical stress at entheses, and altered pharmacokinetics of biologic therapies [21]. Current evidence in psoriatic disease populations supports this concept, demonstrating that targeted weight reduction strategies can significantly improve responses to biologic therapy [22,23]. In the recently reported Tirzepatide in Adults With Moderate-to-Severe Plaque Psoriasis and Obesity or Overweight (TOGETHER-PsO)/PsA trials, combination therapy with the IL-17 inhibitor ixekizumab and the dual glucose-dependent insulinotropic polypeptide/glucagon-like peptide-1 receptor agonist tirzepatide resulted in greater improvements in psoriasis and PsA outcomes compared with ixekizumab alone, suggesting that targeted metabolic intervention may enhance biologic treatment responses in patients with psoriatic disease [24,25]. These findings reinforce the concept that obesity is not merely a comorbidity but a biologically relevant modifier of inflammatory disease pathways and therapeutic outcomes. These findings highlight that treatment response in PsA is shaped not only by inflammatory pathways but also by systemic and metabolic modifiers that influence the dominant disease driver.

## MULTIOMICS APPROACHES AND IMMUNE ENDOTYPING

Multiomics approaches integrating transcriptomic, proteomic, and clinical data have provided new insights into treatment response heterogeneity in PsA. These analyses have started to identify some molecular signatures and immune cell endotypes associated with therapeutic outcomes, highlighting the potential of systems-level profiling to support precision medicine strategies but have yet to be integrated into clinical routine care.

Transcriptomic profiling of peripheral blood and skin fibroblasts has revealed gene expression patterns linked to disease activity, early response, and treatment resistance. Increased transforming growth factor β signalling and persistent expansion of nonclassical monocytes have been associated with treatment resistance, whereas pathways related to platelet activation and Hippo signalling may predict early therapeutic response within the first month of treatment [26]. Integrative analyses combining transcriptomic and proteomic data have also identified circulating protein panels, including S100A8, MMP3, CXCL10, and ACP5, with promising predictive performance for biologic treatment response in PsA (area under the curve > 0.80) [27,28].

High-dimensional immune profiling using mass cytometry and proteomics has further identified immune endotypes such as expansion of memory CD4⁺ T cells, which can be associated with higher musculoskeletal inflammation and reduced response to advanced therapies. These immune phenotypes can appear relatively stable after treatment and can be linked to dysregulated pathways including WNT signalling [29,30].

Despite these advances, translation into clinical practice remains limited. Larger harmonised datasets and international collaborations will be required to validate predictive biomarkers and enable implementation of multiomics-based stratification strategies in routine care [31].

## TOWARDS PHENOTYPE-GUIDED THERAPY IN PsA

Taken together, emerging evidence suggests that personalised therapy in PsA will likely require the integration of multiple layers of information. A conceptual framework for phenotype-guided therapy could include 3 complementary components.

First, clinical phenotyping can identify dominant disease domains and guide the initial therapeutic strategy. Second, imaging biomarkers can distinguish active inflammatory disease from noninflammatory drivers of symptoms and help refine treatment decisions. Third, patient-specific modifiers such as comorbidities, body composition, and psychosocial factors should be systematically incorporated into treatment planning.

Such an integrated approach would represent a shift away from the current sequential treatment paradigm towards a more individualised strategy that targets the specific drivers of disease in each patient.

## TISSUE PROFILING

Tissue profiling, through ultrasound-guided synovial biopsy, and when relevant skin (and, in selected settings, enthesis), is probably the most direct way to see what is actually driving inflammation in an individual tissue in that patient. In principle, this could enable a much more meaningful biological stratification [32], because it captures the immune-stromal pathways operating at the disease site rather than relying only on clinical labels. In practice, though, PsA makes this approach especially hard to scale. The disease is mosaic: for one patient, the dominant process may sit in the synovium, for another in the enthesis, for another in skin/nail, and for others in axial structures, often fluctuating over time. That immediately raises a practical dilemma: which tissue should we sample to best represent “their” disease? And even when the target site is clear, obtaining tissue, particularly repeatedly and from multiple sites, can be logistically difficult, invasive, and not feasible outside specialised centres. As a result, tissue-based stratification risks sampling bias (1 accessible site standing in for a multitissue disease) and will likely remain most realistic in clinical trials and expert hubs, at least until we validate reliable blood/imaging surrogates of tissue endotypes and develop truly scalable, minimally invasive sampling strategies.

In summary, the dominant disease driver of PsA cannot be identified. As PsA is such a complex and heterogeneous disease, different domains have to be considered to identify the dominant disease driver in each patient. The symphony of molecular markers, clinical and imaging phenotypes, and systemic components builds each patient’s individual landscape to identify the most dominant disease driver, see Figure. This landscape has to be re-evaluated over the timely course of the disease, especially when a long-lasting therapy might lose its efficacy.

**Figure:**
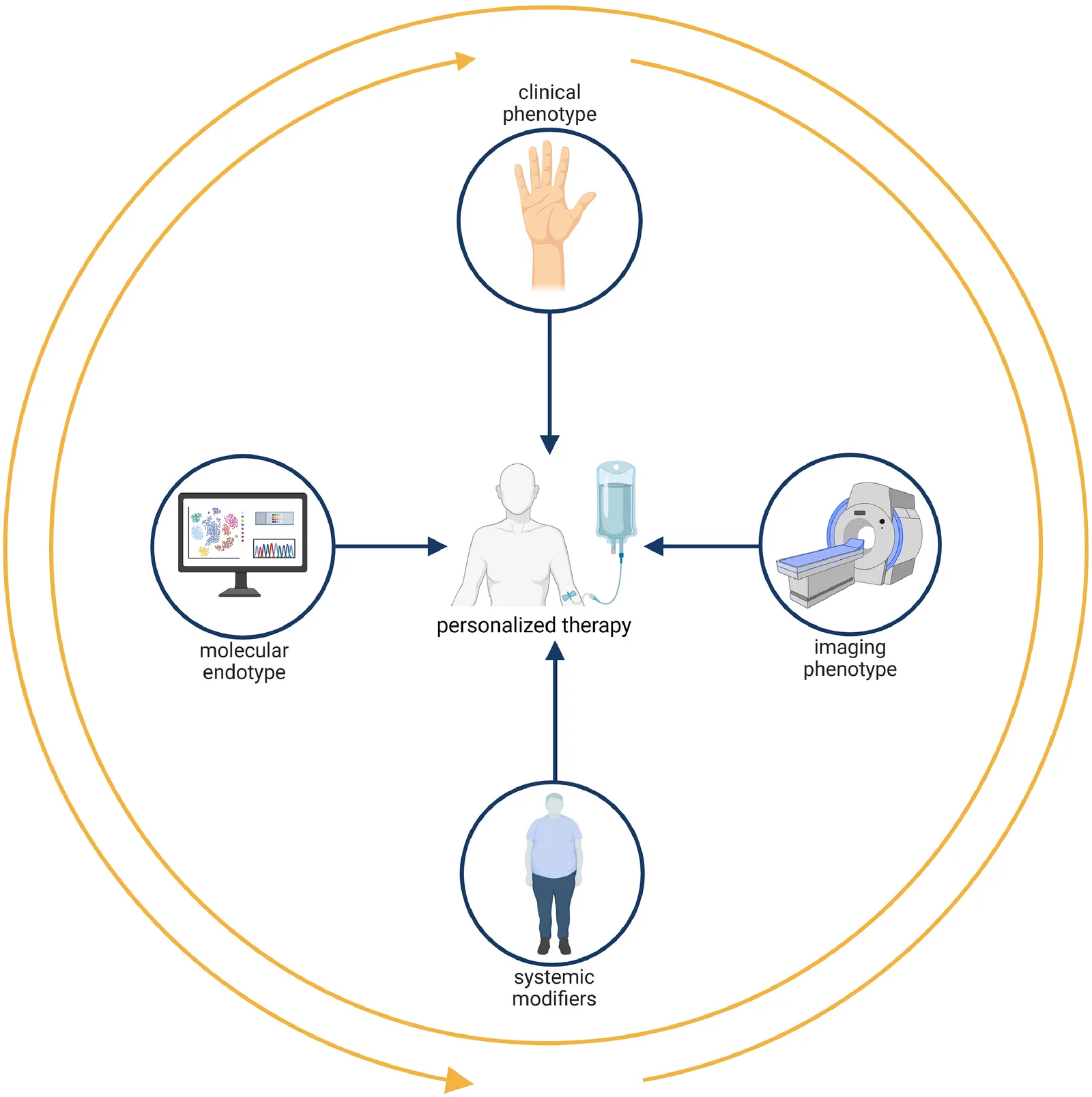
Multilayer framework for driver-guided therapy in psoriatic arthritis. Clinical phenotypes, imaging-defined inflammation, systemic modifiers, and molecular endotypes represent complementary layers that contribute to the identification of the dominant disease driver in an individual patient. Integrating these dimensions may enable driver-guided therapeutic strategies and support precision medicine approaches in psoriatic arthritis.

## LIMITATIONS AND PRACTICAL BARRIERS TO PRECISION MEDICINE IN PsA

Despite its promise, precision medicine in PsA is not yet ready for routine implementation. Advanced imaging, tissue analyses, and multiomics technologies remain costly, infrastructure-dependent and difficult to scale. Many biomarkers have been derived from small or selected cohorts and require independent validation, technical standardisation, and proof of reproducibility. Moreover, molecular signatures may change over time with disease stage, treatment exposure, tissue compartment, and comorbidities. Most importantly, prospective biomarker-driven interventional trials are still lacking. Precision medicine in PsA should therefore currently be regarded as an evolving research framework rather than an established clinical standard.

## RESEARCH AGENDA

Despite substantial progress, several key challenges remain. Future research should focus on the following major priorities:

1. Clinically applicable phenotyping frameworks need prospective validation. While clustering analyses have identified reproducible clinical phenotypes, it remains unclear whether phenotype-informed treatment strategies improve outcomes compared with current trial-and-error approaches.
2. Imaging biomarkers should be integrated into treatment stratification. Musculoskeletal ultrasound and MRI may help distinguish inflammatory from noninflammatory disease drivers and guide therapeutic decision making. Large collaborative initiatives, such as the AutoPiX consortium within the Innovative Health Initiative (IHI2), aim to develop and validate imaging-based biomarkers and automated image analysis approaches to support precision medicine strategies in inflammatory diseases.
3. Multimodal datasets combining clinical, imaging, and molecular data are required to identify disease drivers. Large, harmonised datasets may enable predictive models capable of linking disease drivers to treatment response.
4. Future trials should move beyond mechanism-based comparisons towards phenotype-stratified or driver-guided study designs.

## CONCLUSION

The therapeutic challenge in PsA may no longer be the lack of effective drugs but the lack of tools to identify the dominant disease driver in individual patients at a given point in time. Importantly, these drivers are unlikely to be static: inflammatory activity, structural damage, metabolic and psychosocial modifiers, pain amplification, and tissue-level immune programs may change over the disease course and under therapeutic pressure. Moreover, PsA should increasingly be understood as a systemic immune-mediated barrier disease, in which skin–joint, gut–joint, stromal, and entheseal immune circuits may contribute differently across patients and disease stages. Tissue-resident immune cells, barrier-derived inflammatory signals, and local stromal programs may therefore shape organ-specific manifestations and therapeutic responses. Integrating clinical phenotypes, imaging-defined inflammation, systemic modifiers, and molecular endotypes may enable a shift from empirical drug sequencing towards driver-guided therapy. Such an approach could represent the next step towards precision medicine in PsA.

## Editor disclosure

The peer review process did not involve Editorial Board Members Arnd Kleyer and David Simon, and the editorial decision-making was led by editors who were not involved in the creation of this manuscript.

## CRediT authorship contribution statement

**Thorben Witte:** Writing – review & editing, Writing – original draft, Visualization, Methodology, Formal analysis, Data curation, Conceptualization. **Arnd Kleyer:** Writing – review & editing. **Francesco Ciccia:** Writing – review & editing. **David Simon:** Writing – review & editing, Writing – original draft, Validation, Supervision, Methodology, Formal analysis, Data curation, Conceptualization.

## Competing interests

The authors report no conflicts of interest.
